# Native Valve Endocarditis due to* Veillonella* Species: A Case Report and Review of the Literature

**DOI:** 10.1155/2017/4896186

**Published:** 2017-05-14

**Authors:** Lakshmi Saladi, Cosmina Zeana, Manisha Singh

**Affiliations:** Department of Medicine, Bronx Lebanon Hospital Center, Bronx, NY 10457, USA

## Abstract

*Veillonella* species are fastidious bacteria that have been isolated from skin, dental, and respiratory tract infections and rarely have been implicated in serious infections like meningitis, endocarditis, and osteomyelitis. A 76-year-old woman presented to our hospital with fever, vomiting, and generalized weakness for 3 days. A transthoracic echocardiogram showed a mobile structure on anterior mitral valve leaflet measuring 0.9 cm suggestive of vegetation. Empiric therapy with vancomycin and piperacillin-tazobactam was started with clinical resolution of her symptoms. On day 6, the blood culture drawn at admission grew* Veillonella *species. A transesophageal echocardiogram confirmed a 1.2 × 0.4 cm echo dense structure attached to the left ventricular side of the anterior mitral leaflet. The patient was discharged home after 10 days of inpatient antibiotic therapy and completed 4 weeks of IV ceftriaxone at home without any adverse events. She was reevaluated in the clinic after completion of treatment and repeat blood cultures remained negative. We report the first case of successful treatment of endocarditis due to* Veillonella* species with once daily ceftriaxone.

## 1. Introduction

*Veillonella* species are anaerobic, Gram-negative cocci that are part of the normal mouth, gastrointestinal, and urogenital flora in humans.* Veillonella* species have been isolated from skin, dental, and respiratory tract infections where they are often part of a mixed flora. Rarely,* Veillonella* can cause serious infections like meningitis, endocarditis, and osteomyelitis.

## 2. Case Summary

A 76-year-old woman with medical history of diabetes mellitus, hypertension, chronic hepatitis C with cirrhosis, esophageal variceal bleeding, and stage 4 chronic kidney disease presented to the hospital with fever, vomiting, and generalized weakness for three days.

On physical examination, she was in no apparent distress, a grade 3/6 systolic murmur was audible in the right 2nd intercostal space, lungs were clear to auscultation with equal breath sounds bilaterally, and abdomen was soft and nontender, with normal bowel sounds. She had no focal neurological deficits. Grade 2+ pitting pedal edema was present bilaterally and skin was intact without any rash. The remainder of the examination was unremarkable.

A complete blood count revealed anemia with hemoglobin of 10 g/dL and a white blood cell count of 7500 cell/microliter (81% neutrophils, 6% lymphocytes, and 12% monocytes). Platelet count was 68000 and international normalized ratio (INR) was 1.1. Patient had abnormal renal function with serum creatinine of 2.5. Urinalysis was significant for 150 mg/dL of protein, moderate blood, large leucocyte esterase, and negative nitrite. A transthoracic echocardiogram showed a mobile 0.9 cm structure on the anterior mitral valve leaflet. Empiric therapy with vancomycin and piperacillin-tazobactam was started after 2 sets of blood cultures were obtained. On day 2 of hospitalization, growth of Gram-positive cocci was reported in 1 out of the 2 blood cultures drawn on admission. On day 6, the organism was identified as* Veillonella *species. The patient complained of dysuria during the admission and was diagnosed with urinary tract infection caused by extended spectrum beta lactamase producing* Escherichia coli*. Therefore piperacillin-tazobactam was changed to meropenem to complete 3 days of therapy. A transesophageal echocardiogram confirmed a 1.2 × 0.4 cm echo dense structure attached to the LV side of the anterior mitral leaflet. The isolate was reported sensitive to penicillin and cephalosporins and resistant to metronidazole. At this time, vancomycin was discontinued and meropenem was changed to ceftriaxone. Repeat blood cultures remained negative. The hospital course was complicated by diarrhea due to* Clostridium difficile *which was treated with metronidazole with clinical improvement.

Patient was discharged with a peripherally inserted central catheter after 10 days of inpatient therapy and she completed 4 weeks of IV ceftriaxone at home without any adverse events. She was reevaluated in the clinic after completion of treatment and repeat blood cultures were negative. Follow-up at one year with repeat echocardiogram showed resolution of the vegetation.

## 3. Discussion

*Veillonella* species are small, spherical, Gram-negative cocci that grow under anaerobic conditions on the usual media. They have limited fermentative properties and appear to be harmless saprophytes; though occasionally they may invade the blood stream leading to serious infections like meningitis, endocarditis, and osteomyelitis. 13 species have been identified so far:* V. parvula, V. dispar, V. atypica, V. caviae, V. rodentium, V. ratti, V. criceti, V. montpellierensis, V. denticariosi, V. tobetsuensis, V. magna, V. rogosae, and V. seminalis *[1–3].

There have been 8 prior documented cases of endocarditis with* Veillonella* species as the sole isolate (Table 1). Of these, 4 cases involved prosthetic valves and 4 cases affected native valves.

Interestingly, blood cultures were positive in only 3 of the 8 reported cases [4–6]. In 2 cases the diagnosis was made with growth of the bacteria from valve tissue [7, 8] and in one case the organism was identified with 16sRNA analysis [9]. In our case, 1 out of 2 blood cultures drawn prior to administration of antibiotics were reported positive. If Duke's criteria are strictly applied, this would be categorized as “possible infective endocarditis.”

The optimal antimicrobial treatment of endocarditis caused by* Veillonella* sp. is not standardized. Among the reported cases, antibiotics used include penicillin, ampicillin, first-generation cephalosporin, metronidazole, clindamycin, or aminoglycosides either as monotherapy or in combination [4–8, 10–13]. Penicillin was initially used as the antibiotic of choice [9, 10]. A later study reported high level resistance to penicillin G among oral isolates of* Veillonella* sp. that retained susceptibility to amoxicillin-clavulanate [14]. Among the 8 reported cases of endocarditis, susceptibility data are available in only 3 of the isolates [4, 6, 7]. Reduced penicillin susceptibility was reported in 2 of those [6, 7]. Metronidazole alone has been used in one case of endocarditis caused by* Veillonella* sp. with reduced penicillin susceptibility [7]; only one reported case was treated with a first-generation cephalosporin in combination with gentamicin followed by oral penicillin treatment [8].

In addition to antibiotic therapy, 3 out of the 4 patients with prosthetic valve endocarditis required surgical intervention [4, 5].

The isolate in our patient was susceptible to penicillin (MIC < 0.06), cephalosporins (MIC < 2), ampicillin-sulbactam (MIC < 1), piperacillin, clindamycin, and tetracycline and was resistant to metronidazole. The patient was successfully treated with ceftriaxone alone for 4 weeks.

## 4. Conclusion

The importance of* Veillonella* species as a cause of serious infections including endocarditis has been increasingly recognized. The diagnosis may be difficult due to fastidious nature of the bacterium and there are limited clinical data in the available literature for guiding treatment in these cases. We report the first case of successful treatment of endocarditis due to* Veillonella* species with once daily ceftriaxone.

## Figures and Tables

**Table 1:** Cases of endocarditis due to *Veillonella* species.

	Age/gender	Valve involved	Type of valve	Organism	Antibiotics	Surgery	Microbiologic and clinical cure
(1) [6]	35/M	Mitral	Native	*Veillonella alcalescens*	Penicillin G, sulfadiazine, heparin, and para-aminohippurate for 18 months	No	Yes

(2) [8]	60/M	Aortic	Native	*Veillonella alcalescens*	Cephapirin and gentamicin (2 weeks) and oral penicillin V for 6 months	Yes	Yes

(3) [5]	57/F	Mitral	Prosthetic	*Veillonella dispar*	Ampicillin 2 weeks and clindamycin and metronidazole 2 weeks	Yes	Yes

(4) [12]	NA	Mitral, aortic, and tricuspid	NA	*Veillonella parvula*	Clindamycin	No	Yes

(5) [13]	51/M	Mitral	Prosthetic	*Veillonella alcalescens*	Penicillin G for 6 weeks	Yes	Yes

(6) [4]	56/M	Mitral	Prosthetic	*Veillonella dispar*	Penicillin G for 6 weeks	No	Yes

(7) [7]	49/M	Mitral	Prosthetic	*Veillonella parvula*	Metronidazole for 6 weeks	Yes	Yes

(8) [9]	75/F	Mitral and aortic	Native	*Veillonella montpellierensis*	Amoxicillin (6 weeks) and gentamicin (3 weeks)	No	Information not available

(9) Present work	76/M	Mitral	Native	Veillonella	Ceftriaxone for 6 weeks	No	Yes

M: male; F: female; NA: not available.
